# Evaluating occupational exposures of dental nurses: A retrospective study

**DOI:** 10.3389/fpubh.2022.1010531

**Published:** 2022-11-16

**Authors:** Hongmei Yuan, Rui Shi, Wenwen Chen, Ying Ma, Zhiqing Liu, Fan Liu, Jingmei Yang

**Affiliations:** ^1^State Key Laboratory of Oral Diseases, National Clinical Research Center for Oral Diseases, Department of Periodontics, West China Hospital of Stomatology, Sichuan University, Chengdu, China; ^2^Chengdu Workers' Sanatorium of Sichuan Federation of Trade Unions, North Branch of Health Management Center of Sichuan Provincial People's Hospital, Chengdu, China; ^3^State Key Laboratory of Oral Diseases, Department of Infectious Disease, National Clinical Research Center for Oral Diseases, West China Hospital of Stomatology, Sichuan University, Chengdu, China; ^4^State Key Laboratory of Oral Diseases, Department of Nursing, National Clinical Research Center for Oral Diseases, West China Hospital of Stomatology, Sichuan University, Chengdu, China

**Keywords:** occupational exposures, dental nurse, sharp injury, mucous membrane exposures, training course

## Abstract

**Objective:**

The objective of this study was to investigate occupational blood-borne pathogen exposure among dental nurses and their attitudes toward infected patients, as well as the effectiveness of the training course, to provide a scientific basis for improving the quality of safety management in the dental hospital.

**Materials and methods:**

The study was conducted using questionnaires administered from November 2019 to December 2019 in three hospitals in Sichuan Province, China. Frequencies for answers were calculated and presented as percentages.

**Results:**

In total, 257 valid questionnaires were returned. Most (61.9%) nurses stated that they were involved in occupational exposure. Among them, 154 had experienced sharp injuries, and the syringe needle was the most common instrument for injuries (45.8%). Twenty-two individuals had mucosal exposure, and the proportion of eye exposure was the highest (90.9%). Only associations between training and mucosal membrane exposure were found; however, the relevance was weak (*r* = 0.141). Of the participants, 86.4% felt morally responsible for taking care of patients with infectious diseases, and most (92.6%) said they would continue with this career.

**Conclusion:**

Occupational exposure, particularly to sharp injuries, was common in medical care among dental nurses; however, vocational training had little effect on their incidence. As dental nurses still have positive attitudes toward patients with infectious diseases, more effective training should be conducted.

## Introduction

Occupational exposure is defined as a situation in which healthcare workers are exposed to harmful substances or pathogens of infectious diseases during the process of diagnosis, treatment, and nursing (1). Previous studies have shown that the widespread prevalence of blood infectious diseases is a major risk factor for the transmission of blood-borne pathogens when occupational exposure has occurred, including hepatitis B virus (HBV), hepatitis C virus (HCV), human immunodeficiency virus (HIV), and *Treponema pallidum* (TP) infection (2–5). The government's official statistics in China reported 1,847,230 cases of blood-borne diseases in 2021. The reported incidence rate was 1.3103/100,000, and the mortality rate was 1.43/100,000, an increase of 6.4 and 3.2%, respectively, compared with 2020. The latest statistical report showed that HIV was ranked in the top five disease causes of mortality in China, and hepatitis was also ranked in the top five for morbidity and mortality (6).

Generally, nurses are involved in frontline work; they directly participate in medical treatment, and the quality of their work is related to patients' medical safety, therapy, and recovery (7). Previous studies have shown that nurses have a higher occupational exposure rate than doctors (8, 9). The job of dental nurses is particular because they are exposed to many special sharp instruments, such as endodontic files and ultrasonic tips. Meanwhile, oral operations result in the spatter of many aerosols and saliva drops, which may greatly increase the risk of occupational exposure (10). In this special period, dental practitioners also have a high risk of being exposed to COVID-19 owing to their close contact with saliva drops and aerosols (11).

Previous studies have predominantly focused on the occupational exposure of medical students and dentists in oral clinics (3, 12). In a study focused on nurses, Alanko et al. (13) found that about 41% had reported work-related dermatitis due to occupational exposure; however, few studies have explored the exposures of sharp injuries and mucous membranes. Therefore, this study aimed to investigate the frequency and details of dental nurses' occupational exposure, to assess their attitudes toward infected patients, and to investigate their decisions regarding future careers. In addition, this study explored whether vocational training could decrease the incidence of occupational exposure to improve occupational exposure prevention and control measures.

## Materials and methods

This study was approved by the Institutional Review Board of the West China Hospital of Stomatology, Sichuan University (WCHS-IRB-CT-2022-298). Informed consent was obtained from all participants. This questionnaire was pretested in a pilot study group (*n* = 10) and evaluated in terms of the subjects' understanding and language skills. Based on their replies, we refined the questions and reformulated the questionnaire to make it more suitable for dental nurses (Supplementary material 1). This survey was conducted with 300 dental nurses in three hospitals, including the West China Hospital of Stomatology of Sichuan University (in Chengdu), Mianyang Stomatological Hospital (in Mianyang), and the Department of Stomatology, Hospital of Chinese Traditional Medicine in Meishan (in Meishan), from November to December 2019.

The questionnaire consisted of 14 questions, investigating (1) dental nurses' general characteristics, such as working years, educational background, HBV vaccination immunization, and experience of vocational course, (2) exposure events, including the type and timing of occupational exposure and the sharps and fluids that caused occupational exposure, (3) occupation expectations and attitudes toward patients with infectious diseases. Questionnaires were distributed to dental nurses in these three hospitals, and participation was anonymous and voluntary.

The data were analyzed using SPSS version 20.0 (IBM, USA). Frequencies were calculated and presented as percentages. Comparisons between the data groups were performed using the chi-squared test, with a significance level of *p* < 0.05. Spearman's correlation coefficient was performed to analyze the relationship between the vocational training course and occupational exposure.

## Results

A total of 291 nurses finished the questionnaires (response rate: 97%), of which 34 forms which were incomplete were excluded from the analysis. The numbers and characteristics of the participants are listed in Table 1. Most of the nurses (90.7%) had received vocational training before or during work, and about half (47.5%) were tested for blood-borne pathogens every year. Meanwhile, 232 of the total had received HBV vaccines.

**Table 1:** Basic characteristics of the study participants (*n* = 257).

**Features**	***N*** **(%)**
**Basic information**	
**Educational background**	
Master degree or above	2/257 (0.8)
Bachelor degree	135/257 (52.5)
College degree	120/257 (46.7)
College degree or below	0/257 (0)
**Length of service**	
≤1 year	119/257 (46.3)
1 ≤ 5 years	50/257 (19.5)
5 ≤ 10 years	61/257 (23.7)
≥10 years	27/257 (10.5)
**Overall self-impression**	
**Have you taken the training course on occupational exposure?**	
Yes	233/257 (90.7)
No	24/257 (9.3)
**Have you received the HBV vaccine?**	
Yes	232/257 (90.3)
No	16/257 (6.2)
Not clear	9/257 (3.5)
**Are you tested for HBV, TP, and HIV every year?**	
Yes	122/257(47.5)
No	135/257(52.5)

HBV, hepatitis B; TP, Treponema pallidum; HIV, human immunodeficiency virus.

**Figure 1:**
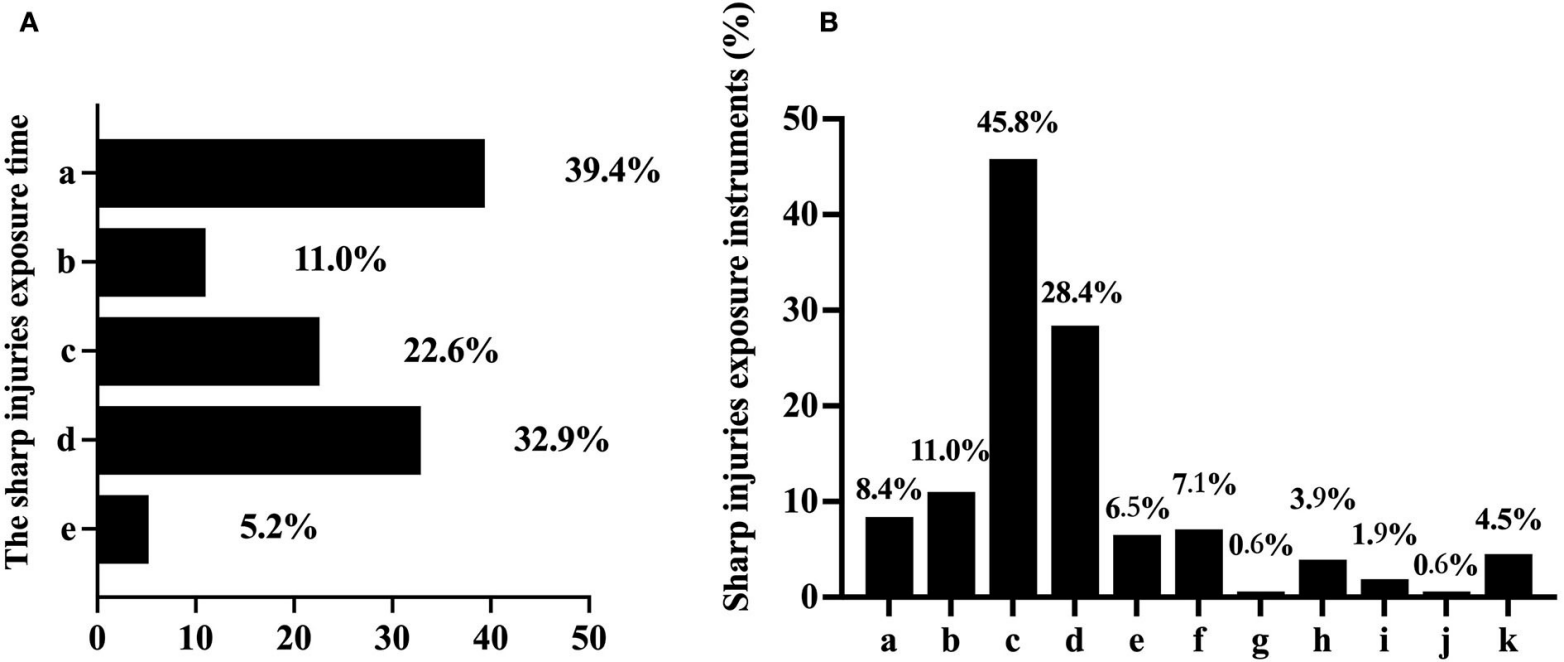
The basic situation of sharp injury exposures (%). **(A)** The exposure time of sharp injuries; a. preparing sharp objects; b. transferring sharp objects; c. operation; d. discarding waste; e. unclear. **(B)** Instruments which may induce sharp injuries: a. scalpel; b. suture needle; c. syringe needle; d. endodontic file; e. ultrasonic tip; f. drilling needle; g. gracey curette; h. periodontal probe; i. barbed broach; j. irrigation needle; k. ligature wire.

Table 2 shows the rate of occupational exposure among dental nurses. Of the 257 cases, 159 were involved occupational exposure, accounting for 61.9%. Sharp injuries accounted for 155 cases and involved exposure to instruments, including scalpels, suture needles, syringe needles, and barbed broaches. Twenty-two nurses (8.6%) had experienced mucous membrane exposure. Figure 1 shows the number of sharp injuries. These events occurred before, during, and after the surgery. The most common time point was the preparation of sharp objects (39.4%). Exposure also occurred at the point of waste disposal (32.9%), followed by the process of operation (22.6%), and the point of transferring sharp objects (11.0%). Of these, 5.2% of them even did not know when they experienced sharp injuries. Of the 155 cases of sharp injuries, the syringe needle was the most common causative instrument of injuries (45.8%). Endodontic files (28.4%), suture needles (11%), scalpels (8.4%), drilling needles (7.1%), and ultrasonic tips (6.5%) were also common (Figure 1).

**Table 2:** Number (percentage) of participants who have experienced occupational exposure (*n* = 257).

**Features**	***N*** **(%)**
**Have you ever experienced occupational exposure?**	
Yes	159/257 (61.9)
No	98/257 (38.1)
**Have you ever experienced mucous membrane exposure?**	
Yes	22/257 (8.6)
No	235/257 (91.4)
**Have you ever experienced sharp injuries exposure?**	
Yes	155/257 (60.3)
No	102/257 (39.7)

**Figure 2:**
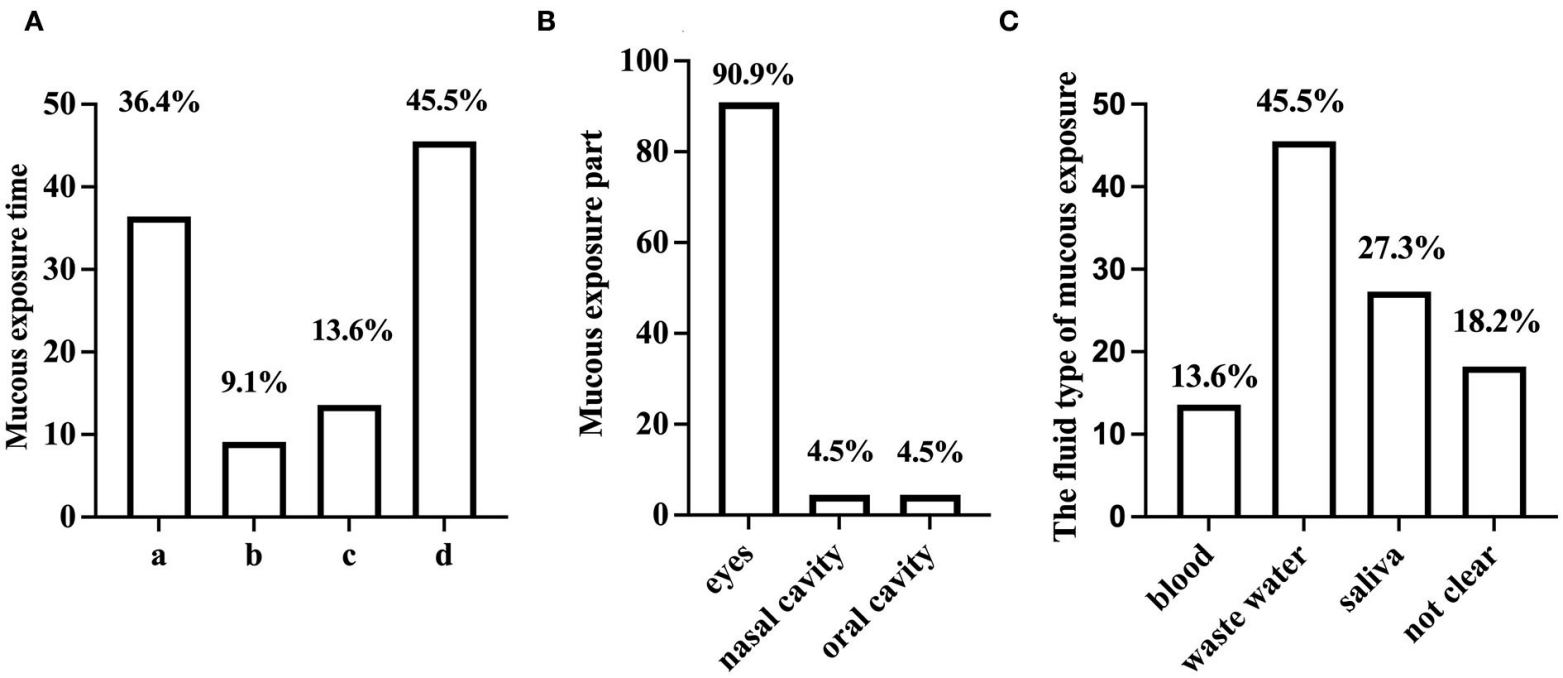
The basic situation of mucous membrane exposure (%). **(A)** The exposure time of the mucous membrane; a. irrigation; b. patients talking; c. splash during the operation; d. discarding waste. **(B)** Mucous membrane exposure. **(C)** The fluid type of mucous membrane exposure.

Figure 2 shows the exposure time, exposure part, and pollutant exposure of the mucous membrane. Among the mucous membrane exposures, the proportion of eye exposures was much higher (90.9%) than in the nasal (4.5%) or oral cavity (4.5%). Exposure occurred mainly during clearing up the instruments (45.5%) and irrigation in patients (36.4%). Therefore, waste fluid (45.5%) and saliva (27.3%) were the main contaminants in mucosal exposure. Interestingly, splashing during the operation (13.6%) was not the main factor of mucosal exposure.

Of the participants, 86.4% felt morally responsible for caring for patients with infectious diseases. Furthermore, 80.5% did not feel scared of this career, even with knowledge of the risk of occupational exposure, and most (92.6%) stated that they would continue with this career (Table 3).

**Table 3:** Occupational expectation (*n* = 257).

**Features**	***N*** **(%)**
**Are you willing to take care of patients with blood-borne pathogens?**	
Yes	222/257 (86.4)
No	35/257 (13.6)
**Are you scared of this career because of the occupational exposure?**	
Yes	50/257 (19.5)
No	207/257 (80.5)
**Have you ever thought about giving up this career because of the**	
**occupational exposure?**	
Yes	19/257 (7.4)
No	238/257 (92.6)

No association was found between vocational training and sharp injuries (Table 4), although a relationship between training and mucous membrane exposure was found (*p* < 0.05). Meanwhile, the results showed that the occurrence of mucous membrane exposure could be influenced by work experience (*p* < 0.01).

**Table 4:** Correlation between nurses' characteristics and occupational exposures.

	**Educational Background**	**Length of service**	**Training course on occupational exposure**
Occupational exposure	−0.038	0.025	0.059
Sharp injuries exposure	0.036	−0.016	−0.069
Mucous membrane exposure	−0.088	−0.161^**^	0.141^*^

* p < 0.05,

** p < 0.01.

## Discussion

Occupational exposure may have side effects on medical staff's psychological problems, bringing about a socioeconomic burden (14–16). In our study, more than 60% of dental nurses experienced occupational exposure, while 90% had already participated in the training course before or during work. No association was found between training and sharp injuries (Table 4), although a relationship between training and mucous membrane exposure was found (*p* < 0.024). Meanwhile, the data showed that the occurrence of mucous membrane exposure could be influenced by working experience (*p* < 0.01). After taking a training course on occupational exposure and the accumulation of working experiences, nurses may be more properly able to use personal protective equipment that can effectively prevent waste fluid or saliva drops from contacting the faces or skin (17). In our study, waste fluid (45.5%) and saliva (27.3%) were the two main contaminants associated with mucosal exposure. Regarding sharp injuries, we did not find a significant correlation between work experience and sharp injuries. The data revealed that syringe needles (45.8%), endodontic files (28.4%), and suture needles (11%) were the top three causative agents of sharp injuries. Needlestick injury was still the most common sharp injury, which agrees with the result of a previous study (18). These oral instruments are so slender that they puncture personal protective equipment easily; therefore, the common use of personal protective equipment sometimes cannot avoid sharp injuries effectively.

Of the 257 valid returned questionnaires, 159 nurses stated that they had experienced occupational exposure, while 155 had experienced sharp injuries, accounting for 60.3% of the total number. This was slightly higher than the results reported in Nigeria (18). This increasing ratio might be related to the development of dental instruments, such as high-speed rotating engine—driven NiTi endodontic files, which are widely used in root canal surgery. This commonly used instrument could not be stopped immediately when the treatment was finished, which might add to the risks of sharp injuries (19). In regular clinical work, the structures of most oral instruments, such as endodontic files, suture needles, scalpels, drilling needles, and ultrasonic tips, are also sharp (20), which may increase the risk of injury during exposure. The syringe needle was also the most common instrument that led to dental nurses' injuries (45.8%); this result was similar to that of a previous study (21). Syringes are often used to administer local anesthesia or during drug injections for mucosal diseases. The pain caused by injection causes patients to move, resulting in an increase in occupational exposure (22). Furthermore, syringes used in oral treatment are sharper than most others because of the fragility of the mucous membrane; this factor may be easily overlooked during rushed clinic work. These may also explain the high proportion of needlestick injuries in occupational exposure. In addition, when anesthesia was required during the treatment, the nurses sometimes helped the dentists recap the needle cap, which inevitably led to exposure. In addition, the narrow space for oral operations also increases the risk of exposure (23).

Our results showed that vocational training did not reduce the rate of occupational exposure. The possible reasons for this are as follows: First, most of them did not pay sufficient attention to the risk of infectious disease in this high-risk environment, and only half of the respondents (47.5%) underwent annual blood testing for infectious disease, although almost all underwent the training course. In total, 232 of all patients had been injected with HBV vaccine before, which may reduce the risk of HBV infection. However, HBV antibody levels may decrease over time, and the protective effect on the human body is weakened (24). Therefore, regular blood testing is essential. Meanwhile, the data showed the most common exposure time (39.4%) was during the preparation of sharps before treatment. At this moment, nurses may regard the instruments as sterile and therefore of low risk, resulting in insufficient attention being paid. In addition, they may use sharp instruments directly instead of tweezers, which increases exposure risks. Second, the existing training mode was not sufficiently effective to reduce occupational exposure (25). The knowledge conveyed in the regular teaching mode is often boring and difficult to understand and is not synchronized with clinical work. Therefore, although most participants underwent the training course, they also had a high rate of sharp injuries. Incorrect separation of the syringe and needle during needle recapping is considered as the most important risk factor associated with sharp injuries (25–27). Furthermore, 5.2% of nurses stated that they did not even know when they experienced sharp injuries. Not knowing when the injury occurred may have resulted in incorrect or insufficient post-exposure handling, which would directly increase the risks of blood-borne pathogen transmission. Heavy clinical work may be another reason why nurses had no time to pay sufficient attention to correct operations, resulting in them ignoring the training contents (28–30).

Mucous membrane exposure is another common occupational exposure. The percentage of individuals who had experienced mucous membrane exposure (8.6%) was far lower than that of individuals with sharp injuries (61.9%) in our study. This might suggest that the necessary personal protective equipment was used to a greater extent. Eye exposure was the most common mucous membrane exposure in our study, accounting for 90.9% of occurrences. This indicated that most nurses did not use protective equipment to protect eyes (14). During treatment, the eyes require constant attention during the operation, and unprotected eyes are likely to be exposed to unpredictable liquid splashes in different directions. As the vision of goggles and face shields is blocked, some nurses may take them off to flexibly cooperate with the dental operations, which greatly increased the risks of mucous membrane exposures (31). The exposure rates of the nasal cavity (4.5%) and oral cavity (4.5%) were relatively low, probably because of the use of masks. Rozanska et al. (1) found that occupational exposure rates decreased with working experience, but increased again after 5 years of working. Therefore, up-to-date training is important throughout the entire career period.

Although the rate of occupational exposure was high in the dental nurse group, we were glad to see that most did not refuse to treat individuals with infectious diseases. They did not feel scared of this career and stated that they would continue in the same field (32, 33). Therefore, training courses on occupational exposure should put this theory into practice and help nurses to improve their awareness to reduce the risk of occupational exposure. It is also necessary to establish standardized operations. For example, it is recommended to use double gloves, cover the needle cap with one hand, and rigorously use protective equipment (34, 35). Furthermore, hospital administration should encourage employees to perform blood tests regularly and to improve occupational exposure report systems (36, 37). Only with the efforts made in a variety of aspects can we reduce the rate of occupational exposure and improve the business confidence of medical staff.

Unfortunately, this survey was only conducted in Sichuan Province, meaning that selection bias and regional specificity are both limitations of this research. Thus, multi-center surveys should be conducted to verify the details of the occupational exposure of dental nurses.

## Conclusion

Occupational exposure, particularly to sharp objects, is a serious problem for dental nurses performing medical care. We found that vocational training had little effect on sharp injuries; however, it could reduce the incidence of mucous membrane exposure to some extent. Despite the high risk of occupational exposure, dental nurses still had positive attitudes toward patients with infectious disease. Therefore, diverse and targeted vocational training is imperative to improve the safety awareness of dental nurses and to reduce the risk of occupational exposure.

## Data availability statement

The original contributions presented in the study are included in the article/Supplementary material, further inquiries can be directed to the corresponding authors.

## Ethics statement

This study was approved by the Institutional Review Board of the West China Hospital of Stomatology, Sichuan University (WCHS-IRB-CT-2022-298). The patients/participants provided their written informed consent to participate in this study.

## Author contributions

HY, RS, and JY had full access to all data used in the study and took responsibility for the integrity of the data and the accuracy of the data analysis. HY, ZL, FL, and JY designed the study. HY, ZL, and JY developed and tested the data collection forms. HY, YM, and WC acquired, conducted, and interpreted the data. HY, RS, FL, and JY drafted the manuscript. All authors critically revised the manuscript and read and approved the final manuscript.

## Funding

This study was funded by the Sichuan Science and Technology Program (No. 2022NSFSC1521).

## Conflict of interest

The authors declare that the research was conducted in the absence of any commercial or financial relationships that could be construed as a potential conflict of interest.

## Publisher's note

All claims expressed in this article are solely those of the authors and do not necessarily represent those of their affiliated organizations, or those of the publisher, the editors and the reviewers. Any product that may be evaluated in this article, or claim that may be made by its manufacturer, is not guaranteed or endorsed by the publisher.
