# Supplementary material for: Evaluating occupational exposures of dental nurses: A retrospective study
Source: Front Public Health. 2022 Nov 16;10:1010531. doi: 10.3389/fpubh.2022.1010531 (PMC9708719; doi:10.3389/fpubh.2022.1010531)
Supplement: Supplementary file 1 [file Data_Sheet_1.PDF]

No：

# Questionnaire of Occupational Exposure

The survey was conducted to improve the quality of hospital safety managements, and strengthen the occupational protection awareness of dental nurses. Please fill in the forms according to your actual situation.

Gender:

Age:

Educational background:

Years of working:

Department:

1. Have you taken the training course on occupational exposure?

A. Yes

B. No

2. Have you received the HBV vaccine?

A. Yes

B. No

C. Not sure

3. Are you tested for HBV, HIV and TP every year?

A. Yes

B. No

4. How many times did you suffer occupational exposures

A. 0

B. 1

C. 2

D. ≥3

5. Have you ever suffered sharps injuries during work before?

A. Yes

B. No

6. Which instruments caused your occupational sharp injuries? (multiple choices)

A. Scalpel

B. Suture needle

C. Syringe needle

D. Endodontic file

E. Ultrasonic tip

F. Drilling needle

G. Gracey scaling instrument
H. Periodontal probe
I. Barbed broach
J. Irrigation needle
K. Ligature wire
L. Others ___

7. When you got the sharp injuries?
A. The timepoint of preparing sharp objects
B. The timepoint of transferring sharp objects
C. The timepoint of operation
D. The timepoint of discarding waster
E. Unclear

8. Have you ever experienced the mucous membrane exposure?
A. Yes
B. No

9. Which part the mucous membrane exposure occurred? (Multiple choice)
A. Eyes
B. Nasal cavity
C. Oral cavity

10. What is the fluid type lead to the mucous membrane exposure?
A. Blood
B. Waste water
C. Saliva
D. Not clear

11. When mucous membrane exposure occurred?
A. The timepoint of irrigation
B. The timepoint of patients' talking
C. The timepoint of splash during the operation
D. The timepoint of discarding waste

12. Are you willing to take care of the patients with infectious diseases?
A. Yes
B. No

13. Have you ever thought about giving up this career because of the occupational exposure?
A. Yes
B. No

14. Are you scared of this career because of the occupational exposure?

A. Yes
B. No
